# Clinical validation of a capnodynamic method for measuring end-expiratory lung volume in critically ill patients

**DOI:** 10.1186/s13054-024-04928-w

**Published:** 2024-04-30

**Authors:** J. A. Sanchez Giralt, G. Tusman, M. Wallin, M. Hallback, A. Perez Lucendo, M. Sanchez Galindo, B. Abad Santamaria, E. Paz Calzada, P. Garcia Garcia, D. Rodriguez Huerta, A. Canabal Berlanga, Fernando Suarez-Sipmann

**Affiliations:** 1 Department of Intensive Care, Hospital Universitario de La Princesa, Diego de León 62, 28006 Madrid, Spain; 2 Department of Anesthesia, Hospital Privado de Comunidad, Mar del Plata, Argentina; 3 Department of Physiology and Pharmacology (FYFA), C3, Eriksson Lars Group, Karolinska Institute, Stockholm, Sweden; 4 Maquet Critical Care AB, Solna, Sweden; 5 CIBER Enfermedades Respiratorias, Instituto de Salud Carlos III, Madrid, Spain; 6 Hedenstierna Laboratory, Uppsala University, Uppsala, Sweden; 7 Deparment of Radiology, Hospital Universitario de la Princesa, Madrid, España

**Keywords:** End-expiratory lung volume, Mechanical ventilation, Ventilation induced lung injury, Lung strain, Respiratory monitoring, Carbon dioxide kinetics, Volumetric capnography

## Abstract

**Rationale:**

End-expiratory lung volume (EELV) is reduced in mechanically ventilated patients, especially in pathologic conditions. The resulting heterogeneous distribution of ventilation increases the risk for ventilation induced lung injury. Clinical measurement of EELV however, remains difficult.

**Objective:**

Validation of a novel continuous capnodynamic method based on expired carbon dioxide (CO_2_) kinetics for measuring EELV in mechanically ventilated critically-ill patients.

**Methods:**

Prospective study of mechanically ventilated patients scheduled for a diagnostic computed tomography exploration. Comparisons were made between absolute and corrected EELVCO_2_ values, the latter accounting for the amount of CO_2_ dissolved in lung tissue, with the reference EELV measured by computed tomography (EELVCT). Uncorrected and corrected EELVCO_2_ was compared with total CT volume (density compartments between − 1000 and 0 Hounsfield units (HU) and functional CT volume, including density compartments of − 1000 to − 200HU eliminating regions of increased shunt. We used comparative statistics including correlations and measurement of accuracy and precision by the Bland Altman method.

**Measurements and main results:**

Of the 46 patients included in the final analysis, 25 had a diagnosis of ARDS (24 of which COVID-19). Both EELVCT and EELVCO_2_ were significantly reduced (39 and 40% respectively) when compared with theoretical values of functional residual capacity (*p* < 0.0001). Uncorrected EELVCO_2_ tended to overestimate EELVCT with a correlation r^2^ 0.58; Bias − 285 and limits of agreement (LoA) (+ 513 to − 1083; 95% CI) ml. Agreement improved for the corrected EELVCO_2_ to a Bias of − 23 and LoA of (+ 763 to − 716; 95% CI) ml. The best agreement of the method was obtained by comparison of corrected EELVCO_2_ with functional EELVCT with a r^2^ of 0.59; Bias − 2.75 (+ 755 to − 761; 95% CI) ml. We did not observe major differences in the performance of the method between ARDS (most of them COVID related) and non-ARDS patients.

**Conclusion:**

In this first validation in critically ill patients, the capnodynamic method provided good estimates of both total and functional EELV. Bias improved after correcting EELVCO_2_ for extra-alveolar CO_2_ content when compared with CT estimated volume. If confirmed in further validations EELVCO_2_ may become an attractive monitoring option for continuously monitor EELV in critically ill mechanically ventilated patients.

*Trial registration*: clinicaltrials.gov (NCT04045262).

**Supplementary Information:**

The online version contains supplementary material available at 10.1186/s13054-024-04928-w.

## Background

The static lung volume remaining in the lung at end expiration i.e. the end-expiratory lung volume (EELV) has important physiological functions. It not only determines the size of the functional lung in which tidal ventilation is distributed, but also acts as a gas reservoir avoiding fluctuations in oxygen and carbon dioxide blood levels [1]. Even in normal lungs this volume is significantly reduced after the induction of anesthesia [2]. In most pathological conditions such as in the acute respiratory distress syndrome (ARDS) this volume can be critically reduced, being an indicator of the severity of the disease [3]. The reduction in EELV results in a heterogeneous distribution of tidal ventilation, amplifying regional stress and strain [4] in those regions receiving a disproportionally high relative tidal volume. This constitutes one of the main mechanisms of ventilation induced lung injury (VILI) that greatly affects patients’ outcome [5]. Despite its relevance, EELV is rarely assessed in the clinical setting as its measurement has been traditionally challenging. Imaging techniques such as computed tomography and magnetic resonance provide accurate estimations of lung volume but have obvious limitations. Other options include inert-gas dilution or single-multiple breath washout, but these methods are cumbersome and require additional difficult-to-handle equipment and are used mainly in the context of clinical research. Currently there are only two methods available for clinical use: (1) Electrical impedance tomography that provides continuous breath-by-breath monitoring of the relative level of changes in lung aeration which is closely related to EELV [6] and (2) a simplified multiple-breath nitrogen wash in/out method which requires the manipulation of inspired oxygen fraction offering intermittent absolute EELV measurements [7].

The capnodynamic method, based on expired CO_2_ kinetics is a clinically attractive alternative for measuring EELV continuously at the bedside [8]. All it requires is conventional expired CO_2_ sensing at the airway opening and the introduction of a modified breathing pattern that generates cyclic changes in the alveolar concentration of CO_2_. Preliminary experimental and clinical evaluations have resulted in good agreement with gas dilution and plethysmographic reference methods [9, 10]. A salient feature of this method is its ability to measure EELV on a breath-by-breath basis being the first method providing continuous absolute EELV values. The potential advantages of this feature have already been advanced in recent evaluations assessing its ability to characterize the lung and adjust the level of end-expiratory positive pressure (PEEP) during laparoscopic surgery [11, 12].

Until now capnodynamic EELV (EELVCO_2_) has only been assessed in experimental models, healthy volunteers and patients with healthy lungs undergoing general anesthesia. In this study we aimed at validating the bias and limits of agreement of EELVCO_2_ in a mixed population of critically ill patients using high resolution computed tomography as the reference method.

## Materials and methods

This is a prospective validation study performed in one single academic teaching center. The study protocol was approved by the local ethics committee (Comité de ética de la investigación con medicamentos del hospital Universitario de la Princesa, Madrid, Spain, April 11, 2019, Registry number 3723), performed in accordance with the Declaration of Helsinki, and registered at clinicaltrials.gov (NCT04045262, on August 5, 2019). Written informed consent was obtained from patient´s next of kin following local regulations.

Adult patients under passive mechanical ventilation in which a lung CT-scan study was clinically indicated by the attending physician were eligible for the study. Those with hemodynamic instability, severe COPD or emphysema, pneumothorax, bronchopleural fistula and under an extracorporeal CO_2_ removal device were excluded.

Patients were under routine clinical monitoring including invasive arterial pressure, pulse-oximetry, surface ECG and usual ventilatory parameters. All were ventilated with a Servo-i ventilator (Maquet Critical Care, Getinge, Solna, Sweden) on controlled mechanical ventilation, under passive conditions, following a lung protective ventilation strategy. Patients were under conventional sedation with the addition of transient muscle relaxation when needed to ensure passive respiratory conditions during the protocol.

### Predicted functional residual capacity

To obtain an estimated theoretical FRC value for each patient, predicted FRC was calculated by the formula described for the supine position in healthy men: [FRC (l) = 5.48 (height (cm) × 0.01) − 7.05] and women: [FRC (l) = 1.39 (height (cm) × 0.01) − 0.424] [13]

### EELV measured by the Capnodynamic method (EELVCO_2_)

The capnodynamic method combines the analysis of end-expiratory CO_2_ kinetics and the differential Fick principle for CO_2_ [8, 10]. Expired CO_2_ is measured by a mainstream infrared sensor (Capnostat®, Philips Respironics, Philadelphia, PA) placed at the airway opening and volumetric capnograms are constructed by combining this signal with the integrated flow measurement in the Servo-i ventilator. By introducing cyclic changes in the alveolar concentration of CO_2_ the necessary information to solve the capnodynamic equation which follows the mole balance principle for CO_2_ in the lung is obtained:

$${\text{EELVCO}}_{{2}} \cdot \left( {{\text{FACO}}_{{2}}^{{\text{n}}} - {\text{ FACO}}_{{2}}^{{{\text{n}} - {1}}} } \right) = {\text{CO}}_{{{\text{EPBF}}}} \cdot \Delta {\text{t}}^{{\text{n}}} \cdot \left( {{\text{C}}_{{\text{v}}} {\text{CO}}_{{2}} - {\text{CcCO}}_{{2}}^{{\text{n}}} } \right) - {\text{VTCO}}_{{2}}^{{\text{n}}}$$

where *EELVCO*_*2*_ is capnodynamic end-expiratory lung volume (L); *FACO*_*2*_, mean alveolar carbon dioxide fraction obtained from the mid-portion of phase III of the capnogram [14]; *n*, current breath; *n *− 1, previous breath, CO_*EPBF*_, effective pulmonary blood flow (L/min); *∆t*^*n*^, current breath cycle time (min), *C*_*v*_*CO*_*2*_, mixed venous carbon dioxide concentration (Lgas/Lblood); *CcCO*_*2*_^*n*^, pulmonary end-capillary carbon dioxide concentration (Lgas/Lblood) derived from the alveolar fraction of CO_2_ and the CO_2_ dissociation curve as proposed by Capek et al. [15]; *VTCO2*^*n*^, volume (L) of carbon dioxide eliminated by the current, n^th^, breath obtained by volumetric capnography. The mole balance of the equation is established between the tidal difference of CO_2_ content between two consecutive breaths (left side) and the amount of CO_2_ supplied to the lung by the blood stream minus the amount of CO_2_ eliminated from the lung (right side). Just a small difference in alveolar CO_2_ of only 2–3 mmHg is needed to obtain consistent values. To this end the ventilator applies a modified cyclic breathing pattern in which short expiratory holds are added to the final 3 out of 9 consecutive mechanical breaths. To calculate EELVCO_2_ the modified breathing pattern must be stable and the patient under passive ventilatory conditions. Assuming that pulmonary blood flow and mixed venous content of CO_2_ (CvCO_2_) remain constant during a 9-breath measurement cycle the three unknowns of the equation, EELVCO_2_, CvCO_2_ and the effective (i.e. non-shunted) pulmonary blood flow can be obtained. These nine breaths create nine capnodynamic equations that are solved by the least square method in which measured FACO_2_ data are fitted with those obtained from an ideal one compartment lung model. The capnodynamic equation system is applied continuously, breath-by-breath, where every new breath is replacing the first one in the sequence. This results in a continuous breath-by-breath monitoring of EELVCO_2_ and CO_EPBF_.

### Corrections

Due to the specific particularities of CO_2_ as a tracer gas for measuring lung volume we applied two corrections:

1. As CO_2_ kinetics are measured in expired gases, the volume corresponding to the larger airways (i.e. airway dead-space) was obtained and subtracted from the final EELVCO_2_ measurement. Gas filling larger airways was also discarded from EELVCT by manually excluding the larger visible airways in each analyzed CT slice.
2. Total lung CO_2_ volume is composed by 3 principal components: alveolar, tissue and blood CO_2_. As the traditional inert-gas measured EELV refers only to the alveolar gas, EELVCO_2_ has a tendency to overestimate EELV. To account for the tissue and blood CO_2_ stores we introduced a previously described correction factor that considers this amount to be around 20% of total lung CO_2_ [16, 17]: EELVCO_2_ corr = 0.8 × EELVCO_2_. See Additional file 1 for a more detailed explanation.

### EELV measurement by computed tomography

Quantitative CT volume analysis was used as the reference method. Lung CTs were obtained by the Siemens Somatom Sensation 40 work station and General Electric® Revolution EVO. CT acquisition configuration was as follows: tube voltage 100–120 kV, current 150–578 mA collimation 0.625–1.2 mm, table speed 38–98 mm/seg, pitch 0.96 to 1.5 and slice thickness 0.625 to 5 mm. Lung images were obtained during expiration maintaining the established level of end-expiratory positive pressure by switching to a continuous positive pressure (CPAP) mode, as described below.

Images were analyzed using OsiriX lite, 12.0v (Pixmeo® Geneve, Switzerland). Regions of interest (ROIs) were manually drawn, carefully excluding extra lung-parenchymal structures such as chest wall, mediastinum, hilar vascular structures, major blood vessels, trachea and major bronchi.

The EELV measured by lung CT density analysis (EELV_CT_) was obtained as: ∑ Gas Volume = ∑ [(CT/1000) × Voxel Volume], assuming a lung tissue density similar to that of water.

The obtained total EELV_CT_ included all lung compartments containing air with densities ranging from − 1000 to 0 Hounsfield units. As EELVCO_2_ can, to a certain extent, be considered the volume participating in gas exchange, it was also compared with a more functional CT volume excluding the compartments with densities higher than − 200 HU in which a significant shunt effect is assumed to be present [18].

### Study protocol

After confirming clinical stability and passive ventilatory conditions a lung volume history homogenization maneuver was performed by increasing PEEP to 20 cmH_2_O and driving pressure to 20 cmH_2_O for 1 min. After returning to baseline settings, the capnodynamic breathing pattern was started.

A preliminary 30 min measurement period was performed in the ICU before transferring the patient to the CT facility to test the stability of the signals and for adjustments of the breathing pattern when necessary. During CT transfer patients remained connected to the same ventilator maintaining the breathing pattern. The CT sequence was acquired during expiration by briefly changing to a CPAP mode during the few seconds of the acquisition ensuring to maintain the same PEEP selected by the clinician. After returning to the ICU a final acquisition period of 30 min was performed. To maintain the same conditions, all protocol measurements were performed in the supine position at 0° (mandatory during the CT acquisition). Extreme caution was taken to avoid any disconnection from the ventilator circuit and to alter or affect patient positioning. A new lung homogenization maneuver was performed in case of inadvertent disconnection.

### Data analysis

In this exploratory validation study an arbitrary sample size of 50 patients was selected. All the EELVCO_2_ recordings were analyzed independently by one of the investigators blinded to the acquisition process and to the EELV_CT_ values. A stable, representative period of the recording generally averaging a value over a period of several minutes was used for comparison at the CT facilities before the acquisition or, when the measurement was too noisy during this period, during the 30 min after returning to the ICU. The software includes an error function to discard noisy measurements and only periods in which the error was less than a predefined threshold level were considered for analysis (Additional file 1: Figures S9 and S10). CT scans were analyzed by 2 investigators blinded to the EELVCO_2_ values.

Normality of the data including the differences between the means of the capnodynamic and CT measured volumes was assessed by the Shapiro–Wilk test. The Bland–Altman methodology was used for the comparison between methods obtaining bias to establish the accuracy of the method and limits of agreement (LoA, mean ± 1.96 × standard deviation) to analyze the precision of the method.

Pearson´s correlation was used to establish the correlation between normal distributed variables.

Descriptive values are presented as mean ± standard deviation or median – range depending on their distribution. A *p* value < 0.05 was considered significant. Data were analyzed using the SPSS software package (SPSS statistics, IBM, v25).

Both methods were also compared when subdividing studied patients in those with and without a diagnosis of ARDS.

## Results

**Fig. 1:**
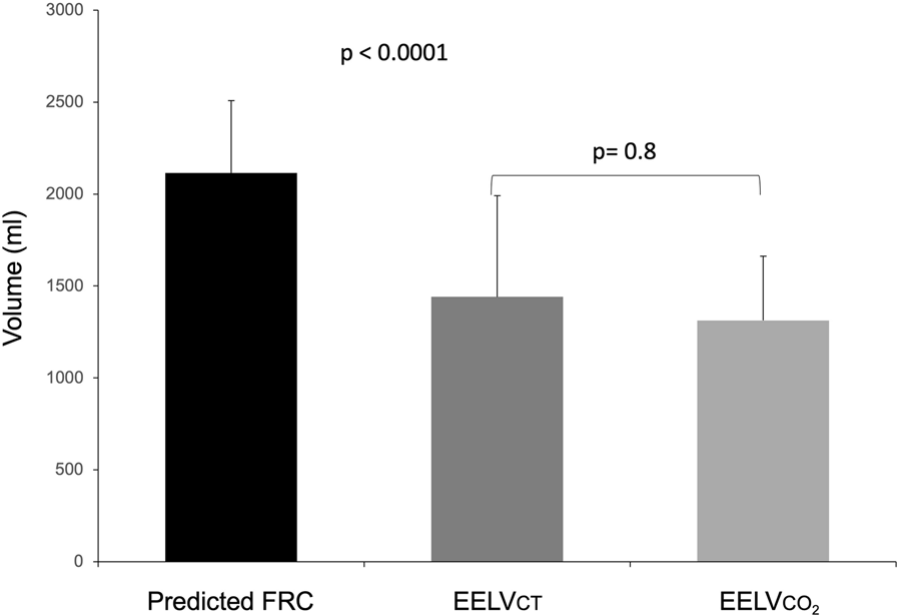
Comparison between theoretical FRC in the supine position, total CT volume and corrected EELVCO2

From the 50 patients studied 4 were excluded from the analysis: three due to previously unknown severe lung emphysema discovered during the CT study and one due to technical problems in which the CO_2_ signal was lost. Thus, a total of 46 patients were included in the final analysis. Table 1 presents the baseline characteristics and Table 2 respiratory, lung mechanics and gas exchange parameters of included patients. As the study was performed mainly during the pandemic, half of the included patients had a diagnosis of COVID-19 related ARDS. Both EELVCT and EELVCO_2_ confirmed a marked decrease in EELV (of 39 and 40% respectively), when compared with predicted normal FRC values (Fig. 1, Tables 1 and 2). In ARDS patients this decrease was more pronounced (Additional file 1: Figure S4) and they presented worse oxygenation and increased dead space.

**Table 1:** Baseline characteristics

Demographics	N (46)
Gender (M/F)	30 (66%)/15(33%)
Age (years)	68 ± 11
Weight (kg)	80 ± 13
Height (cm)	167 ± 9
PBW (kg)	62 ± 10
BSA (m^2^)	1.88 ± 0,17
BMI (kg/m^2^)	28.7 ± 4.1
APACHE II	17.4 ± 6.6
FRC pred (mL)	2167 ± 386
*Diagnosis*	
C-ARDS	25
ARDS	1
Pneumonia	3
Hemoptysis (Lung Cancer)	1
Septic shock	5
Heart failure/Cardiac surgery	7
Haemorrhagic shock	1
Intoxication	2
Encephalitis	1

PBW, predicted body weight; BSA, Body Surface area; BMI, Body mass Index; FRCpred, predicted body weight in the supine position and according to gender (Reference ….)

**Table 2:** Gas exchange respiratory and lung mechanics parameters

	All Patients	Non-ARDS	ARDS	*p* value
	N = 45	N = 20	N = 25	
PaO_2_ (mmHg)	93.6 ± 32.6	111.4 ± 39.2	80.8 ± 17.4	< 0.001
FIO_2_	0.6 ± 0.2	0.5 ± 0.2	0.68 ± 0.21	0.004
PaO_2_/FIO_2_	177.8 ± 87.9	237.6 ± 81.4	132.3 ± 62.2	< 0.001
SaO_2_ (%)	96.8 ± 3.2	97.9 ± 3.0	95.9 ± 3.1	< 0.04
PaCO_2_ (mmHg)	47.8 ± 13.4	43.7 ± 10.1	51.1 ± 14.9	0.07
PACO_2_ (mmHg)	34.1 ± 8.6	35.5 ± 7.6	32.9 ± 9.5	0.34
PETCO_2_ (mmHg)	37.7 ± 9.6	37 ± 9	36.8 ± 10.2	0.78
VD/VT_Enghoff_	0.54 ± 0.13	0.47 ± 0.13	0.58 ± 0.11	< 0.007
VD/VT_Bohr_	0.34 ± 0.09	0.31 ± 0.07	0.36 ± 0.06	0.036
VCO_2_ (ml/min)	240 ± 47	202 ± 46	220 ± 34	0.15
pHa	7.40 ± 0.09	7.41 ± 0.08	7.39 ± 0.11	0.48
HCO_3_^−^ (mmol/l)	29.5 ± 6.5	27.6 ± 4.5	30.8 ± 6.5	0.11
Hb (gr/dl)	9.6 ± 1.8	8.8 ± 1.3	10.3 ± 1.8	0.002
VT (ml)	422 ± 87	427 ± 78	430 ± 89	0.89
VT/kg (ml/kg)	6.9 ± 1.3	7 ± 1.1	7.1 ± 1.5	0.89
RR	20.3 ± 3.5	19.7 ± 3.7	21.6 ± 2.6	0.03
VM (l/min)	8.6 ± 1.8	7.9 ± 1.9	9.1 ± 1.7	0.04
VA (l/min)	6.1 ± 1.2	5.8 ± 1.2	6.2 ± 1.2	0.58
Pplat (cmH_2_O)	26.2 ± 3.7	25.8 ± 3.8	26.8 ± 3.5	0.20
PEEP (cmH_2_O)	10.9 ± 2.9	11.4 ± 3	11.2 ± 3	0.41
PDriving (cmH_2_O)	14.6 ± 4.6	13.8 ± 4.1	14.9 ± 4.9	0.07
Crs (ml/cmH_2_O)	32.7 ± 14.2	34.3 ± 13.8	32.4 ± 15.9	0.97
EELVCT (ml)	1335 ± 573	1440 ± 550	1215 ± 582	0.25
EELVCT _funct_ (ml)	1293 ± 585	1405 ± 554	1208 ± 527	0.17
EELVCO_2_ (ml)	1620 ± 562	1640 ± 436	1605 ± 650	0.84
EELVCO_2corr_ (ml)	1296 ± 450	1312 ± 348	1275 ± 529	0.79

PaO_2_, arterial partial pressure of oxygen; FIO_2_, Fraction of inspired oxygen; SaO_2_, arterial hemoglobin oxygen saturation; PaCO_2_, arterial partial pressure of carbon dioxide; PACO_2_, alveolar partial pressure of carbon dioxide; PETCO_2_, end-tidal partial pressure of carbon dioxide; VD/VT_Enghoff_, physiological dead space according to Enghoff’s formula; VD/VT_Bohr_, physiological dead space according to Bohr’s formula; VCO_2_, volume of CO_2_ elimination per minute; pHa, arterial blood pH; HCO_3_^−^, arterial blood bicarbonate; Hb, hemoglobin concentration; VT, expired tidal volume; VT/kg, expired tidal volume per kg predicted body weight; RR, respiratory rate; VM, minute volume; VA, alveolar ventilation; Pplat, plateau pressure; PEEP, positive end-expiratory pressure; Pdriving, inspiratory driving pressure (computed as Pplat – PEEP); Raw, airway resistance; Crs, respiratory system compliance; EELVCT, Computed tomography end-expiratory lung volume; EELVCT funct, Computed tomography measured functional volume comprising al voxels from – 1000 to – 200 HU EELV_CO2_, Capnodynamic end-expiratory lung volume; EELV_CO2 corr_, Capnodyamic end-expiratory lung volume after correcting for CO_2_ volume contained in the lung. *p* values are calculated for non-ARDS vs ARDS patients

**Fig. 2:**
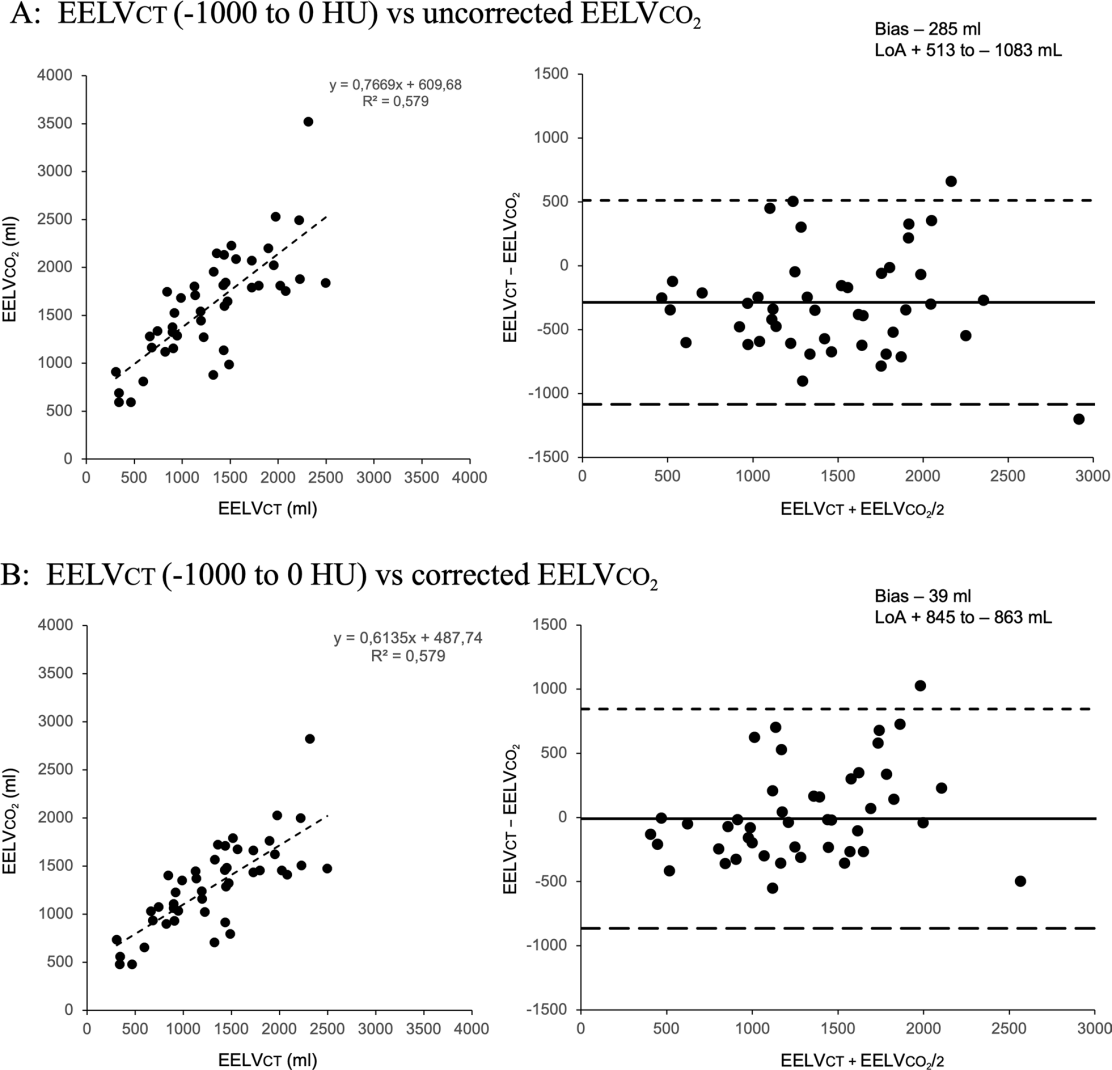
Comparison of total CT volume with uncorrected (**A**) and corrected (**B**) EELVCO_2_

**Fig. 3:**
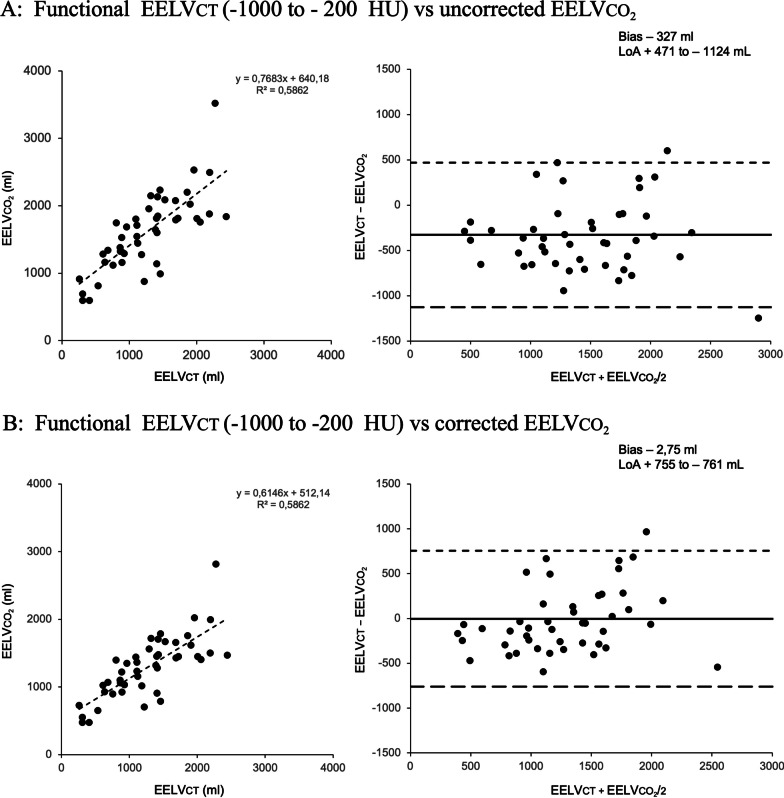
Comparison of functional CT volume with uncorrected (**A**) and corrected (**B**) EELVCO_2_

The mean differences between both methods followed a normal distribution. The method overestimated EELV_CT_ in 39 out of the 46 patients included (85% of the cases). Uncorrected EELVCO_2_ values presented a correlation (r^2^); bias and LoA (95% CI) of 0.58; − 285 and (+ 513 to − 1083) ml when compared with total EELV_CT_ (Fig. 2A) and 0.59; − 327 (+ 471 to − 1124) ml when compared with the functional EELVCT (Fig. 3A). EELVCO_2_ correction for lung CO_2_ content resulted in a correlation; bias and LoA (95% CI) of 0.58; − 39 (+ 845 to − 863) ml for the total and 0.59; − 2,75 (+ 755 to − 761) ml for the functional EELVCT volumes providing a good correction for the method’s overestimation, improving bias and to a lesser extent the limits of agreement (Figs. 2B and 3B). We found that the overestimation of the method was larger at lower lung volumes (Additional file 1: Figure S1) and higher gas-tissue ratios (Additional file 1: Figure S2) which was also confirmed when analyzing the residuals of the regression analysis (Additional file 1: Figure S3).

**Fig. 4:**
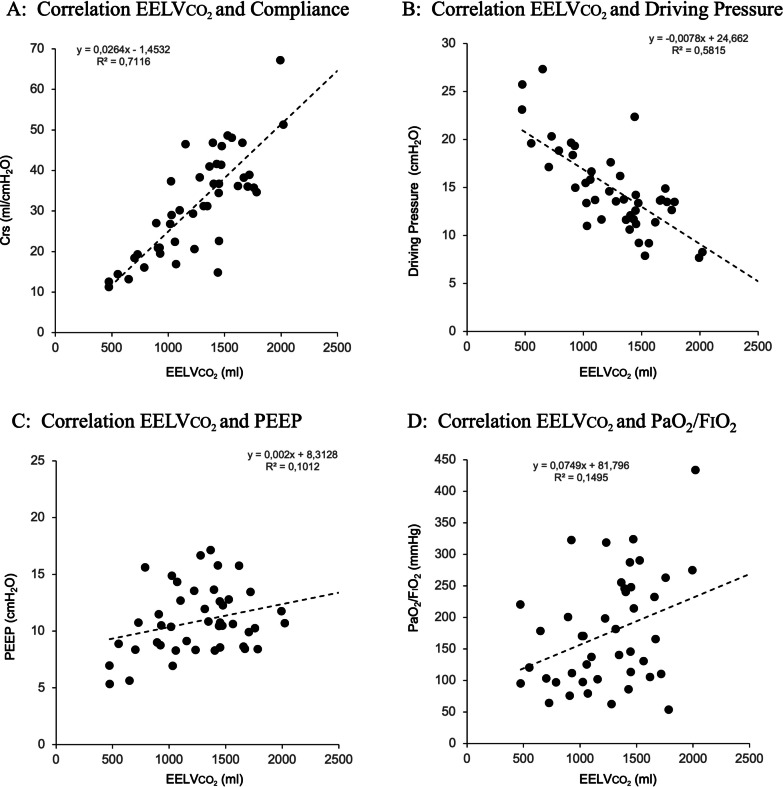
Correlations between corrected EELVCO_2_ with respiratory system compliance (**A**); Driving pressure (**B**); PEEP (**C**) and PaO_2_/FiO_2_ (**D**). EELVCO_2_ was well correlated with respiratory system compliance and driving pressure reinforcing the notion that these two parameters are good indicators of the size of the functional lung volume. However correlation with PEEP and oxygenation was very poor

When comparing non-ARDS with ARDS and patients with the functional EELVCT, differences for the uncorrected values were small indicating a similar performance of the method presenting a correlation, bias and LoA of 0.39; − 235 (631 to – 1100) and 0.69; – 387 (329 to 1123) ml respectively (Additional file 1: Figures S5 and S6). Values after applying the correction factor changed to – 86 (574 to – 746) ml in ARDS patients (Additional file 1: Figure S8) and tended to overcorrect EELVCO_2_ in non-ARDS patients 93 (941 to – 754) ml (Additional file 1: Figure S7) Table 3). EELVCO_2_ correlated well with respiratory system compliance and driving pressure but was unrelated to the level of PEEP or oxygenation (Fig. 4).

**Table 3:** Comparisons between methods: correlations, bias and limits of agreement

	All Patients		Non-ARDS		ARDS	
	Correlation R^2^/Bias (LoA)		Correlation R^2^/Bias (LoA)		Correlation R^2^/Bias (LoA)	
	EELVCT	EELVCT _funct_	EELVCT	EELVCT _funct_	EELVCT	EELVCT _funct_
EELVCO_2_	0.58/− 285 (513 to − 1083)	0.59/− 327 (471 to − 1124)	0.39/− 199 (667 to − 1067)	0.39/− 235 (631 to − 1100)	0.71/− 351 (380 to − 1082)	0.69/− 387 (329 to − 1123)
EELVCO_2 corr_	0.58/− 39 (845 to − 863)	0.59/− 2.75 (755 to − 761)	0.45/128 (975 to − 719)	0.45/93 (941 to − 754)	0.71/− 41 (587 to − 668)	0.73/− 86 (574 to − 746)

LoA, limits of agreement; EELVCT, End-expiratory volume measured by computed tomography (− 1000 to 0 HU); EELVCT funct, Functional End-expiratory volume measured by computed tomography (− 1000 to − 200 HU); EELVCO_2_, End-expiratory volume measured by the capnodynamic method; EELVCO_2 corr_, Corrected end-expiratory volume measured by the capnodynamic method

## Discussion

In this validation study the presented capnodynamic method provided reasonable estimates of end-expiratory lung volume in critically ill patients on mechanical ventilation when compared to the reference volume measured by computed tomography. The method performed with a small bias but large limits of agreement*.* Bias improved after correcting the capnodynamic EELV for the lung’s extra-alveolar CO_2_ content and was similar across different degrees of lung disease.

Using CO_2_ as a tracer gas for lung volume measurements was already considered as early as in the fifties [16]. Advantages include its endogenous production providing a continuous source for measurement without the need for any exogenous gas. Furthermore, it is easy to measure at the airway opening by infrared light absorption, a well-established, cheap, widely available and reliable sensing technique [19]. However, CO_2_ has a higher solubility than most inert gases, it depends on lung perfusion, diffusion across the alveolo-capillary membrane and its distribution in the lung. As mentioned before CO_2_ also enters the lung tissue where it dynamically equilibrates with the alveolar concentration so that its final elimination in exhaled air, the point of measurement, can be affected by several factors such as the ventilation-perfusion balance and the tissue-to-gas ratio in the lung. The foundations of the capnodynamic method to measure EELV were first proposed by Gedeon et al. by introducing small short-lasting changes in the lung’s CO_2_ balance by means of brief inspiratory holds added to the breathing pattern [20]. Later, Brewer et al., described a CO_2_ rebreathing method obtaining good bias and limits of agreement in a first experimental evaluation with only a small overestimation compared to a nitrogen-based method despite applying a correction factor [21]. In a subsequent clinical evaluation in mechanically ventilated patients, bias and limits of agreement of the rebreathing based method were – 20 ± 260 mL compared with nitrogen wash-out EELV [17].

Recently the Capnodynamic method was reformulated and refined. A first comparison of EELVCO_2_ with helium multiple breath washout and CT was made in healthy and lung injured rabbits submitted to three different PEEP levels [22]. EELVCO_2_ reflected the changes in lung volume due to PEEP but overestimated the volume obtained at low PEEP in healthy animals. In a second study in a similar experimental model, they confirmed a systematic overestimation at low PEEP levels in the healthy condition that decreased in injured animals at PEEP > 6 cmH_2_O. Sander et al. studied the influence of changes in cardiac output and different levels of PEEP on EELVCO_2_ in a porcine model. They found that EELVCO_2_ was closely related to EELV measured by sulfur-hexafluoride washout and that it remained stable during changes in PEEP and large changes (> 40%) in cardiac output [9]. In a first clinical evaluation, Öhman et al. found similar bias and limits of agreement for uncorrected EELVCO_2_ values as in the current study using plethysmography in healthy volunteers and multiple breath nitrogen washin-washout in patients under general anesthesia during head-neck surgery [10].

In this study we compared the capnodynamic method with high resolution CT, a frequently used reference method for lung volume studies in ventilated patients [23]. CT performed during expiration precisely measures a one-point anatomical lung volume including all gas containing regions. In this population of patients, the capnodynamic method overestimated EELVCT by a mean value of 285 ml (21,3%). Applying the correction and comparing it with the “functional” EELVCT the average difference decreased to – 3 ml (− 0.23%). The volume overestimation of the method was larger at lower lung volumes (Additional file 1: Figure S1) and at higher tissue-to-gas ratios (Additional file 1: Figure S2) pointing to the importance of the lung tissue CO_2_ component in the overestimation of the method. Limits of agreement were comparatively larger than the ones obtained by other inert gas-based methods such as helium and nitrogen [24] and oxygen [25].

One possible explanation for these wider limits of agreement may be related to the fact that one single measurement point in the CT was compared with a continuous method. For the comparative value of EELVCO_2_ we averaged the value of 20 min recording (corresponding to approximately 400 measurements) during stable (minimal system error) representative conditions. Despite selecting stable periods, in some cases we observed a variability of the EELVCO_2_ of up to ± 300 ml during the sequence recording (Additional file 1: Figure S9). After prolonged on-line observations of continuous EELV monitoring, we are inclined to interpret this as a biological variability representing a true lung behavior rather than random variability of the measurement method. We did not find any differences in lung volume between ARDS and non-ARDS patients (Additional file 1: Figures S3 to S6).

## Limitations

There are some methodological aspects of this study that need to be commented. First, we incurred in a certain selection bias towards more severely ill patients as the criterion for performing a thoracic CT was based on a clinical decision. This would in any case act in favor of the method as it is in this population were monitoring EELV is of particular clinical relevance. Second, all except one ARDS patients had a diagnosis of COVID which has been shown to have a greater amount of lung gas when compared with compliance-matched ARDS patients of other etiologies [26]. Therefore, it is important to highlight that our results may not be generalizable to non-COVID ARDS patients. Third, due to signal instability during CT acquisition and time constraints at the CT facilities, in most patients EELVCO_2_ was analyzed in the immediate post CT period in the ICU. Although patients were kept in the supine position and a new volume history homogenization maneuver was performed in case of accidental disconnections, changes in the surface on which patients were lying or small differences in the overall body position may have accounted for differences in the measured volumes. Fourth, the capnodynamic method measures a lung volume largely composed by the one participating in gas exchange. This functional lung volume is not exactly the same as a static lung volume although it may have a particular monitoring interest in patients on mechanical ventilation. Fifth, we did not measure shunt and thus did not evaluate its influence or correct for its effect on the method. Sixth, the correction factor applied of 0.8 is an estimated average and does not take into account global or regional differences in the tissue-to-gas ratio and its relation to regional perfusion so that it can under or over correct in more borderline conditions.

## Clinical relevance

The capnodynamic method provides the unprecedented possibility to monitor changes in EELV on a continuous breath-by-breath basis, in critically ill mechanically ventilated patients. This can have interesting clinical monitoring implications. First, it can quantify the size of the baby lung and follow its changes over time. This can become a maker of severity of lung disease with prognostic value. Second, it offers the option to monitor breath-by-breath changes in the global dynamic lung strain, one of the principal mechanisms of ventilation induced lung injury [27], at a given moment or in response to changes in the lung condition or ventilator settings. Third, it can help improve the selection of a protective tidal volume. Instead of adjusting VT to the predicted body weight, a fixed value based on the anatomical size of the lung, it could now be adjusted to the functional size of the lung adapting to its dynamic changes during the course of the mechanical ventilatory support. Fourth, changes in EELV can be used to improve the monitoring of the response to PEEP or lung recruitment. It can inform about lung recruitability, helping to identify patients with a higher potential for recruitment and thus more responsive to PEEP. Bias for the corrected volume was low but limits of agreement were high reducing the precision of the method. How this lower precision affects the overall clinical performance EELVCO_2_ or whether the performance can be in part compensated by the continuous nature of the measurement in critically ill patients will have to be assessed in future evaluations. In a previous clinical study, EELVCO_2_ showed excellent trending ability with concordance rates of 100% with the reference methods [10]. In two more recent studies the method demonstrated a good performance helping to select a protective level of PEEP in adult patients [11] and children [12] submitted to laparoscopic surgery.

## Conclusions

In a mixed population of critically ill patients on passive mechanical ventilation the capnodynamic method provided reasonable estimates of EELV in both ARDS and non-ARDS patients. The capnodynamic EELV corrected for extra-alveolar CO_2_ presented a very good bias but large limits of agreement similar to the ones obtained in previous validations in non-critically ill patients. In the current evaluation almost all ARDS patients were COVID related. If these results can be confirmed in further evaluations including other ICU patient populations and a larger proportion of non-COVID related ARDS patients, this method can become a promising bedside tool for the continuous monitoring of EELV.

### Supplementary Information

**Additional file 1**. Supplementary materials and figures.

## Data Availability

The data that support the findings of this study are not openly available due to reasons of sensitivity and are available from the corresponding author upon reasonable request. Data are located in controlled access data storage at Hospital Universitario de la Princesa (Madrid, Spain).
