# Correction to: Clinical validation of a capnodynamic method for measuring end‑expiratory lung volume in critically ill patients

**DOI:** 10.1186/s13054-024-04984-2

**Published:** 2024-06-14

**Authors:** J. A. Sanchez Giralt, G. Tusman, M. Wallin, M. Hallback, A. Perez Lucendo, M. Sanchez Galindo, B. Abad Santamaria, E. Paz Calzada, P. Garcia Garcia, D. Rodriguez Huerta, A. Canabal Berlanga, Fernando Suarez‑Sipmann

**Affiliations:** 1 Department of Intensive Care, Hospital Universitario de La Princesa, Diego de Leon 62, 28006 Madrid, Spain; 2 Department of Anesthesia, Hospital Privado de Comunidad, Mar del Plata, Argentina; 3 Department of Physiology and Pharmacology (FYFA), C3, Eriksson Lars Group, Karolinska Institute, Stockholm, Sweden; 4 Maquet Critical Care AB, Solna, Sweden; 5 CIBER Enfermedades Respiratorias, Instituto de Salud Carlos III, Madrid, Spain; 6 Hedenstierna Laboratory, Uppsala University, Uppsala, Sweden; 7 Deparment of Radiology, Hospital Universitario de La Princesa, Madrid, Spain

**Correction to: Critical Care (2024) 28:142 (2024)** 10.1186/s13054-024-04928-w

Following publication of the original article [1], the authors identified an error within the funding project number in the Funding section.

The Funding section currently reads:

**Funding**

This study has been funded by Instituto de Salud Carlos III through the project “FIS.20/01548” (Co-funded by European Regional Development Fund/European Social Fund. “A way to make Europe”/“Investing in your future”). Project “FIS.20/01548”, funded by Instituto de Salud Carlos III and co-funded by European. Union (ERDF/ESF, “A way to make Europe”/“Investing in your future”). Funding: ISCIII (“FIS.20/01548”), co-funded by ERDF/ESF, “A way to make Europe”/“Investing in your future”).

The Funding section should read:

**Funding**

This study has been funded by Instituto de Salud Carlos III through the project “PI20/01548” (Co-funded by European Regional Development Fund/European Social Fund. “A way to make Europe”/“Investing in your future”). Project “PI20/01548”, funded by Instituto de Salud Carlos III and co-funded by European. Union (ERDF/ESF, “A way to make Europe”/“Investing in your future”). Funding: ISCIII (“PI20/01548”), co-funded by ERDF/ESF, “A way to make Europe”/“Investing in your future”).

The Funding section has been updated in this correction and the original article [1] has been corrected.
