# Supplementary material for: Clinical validation of a capnodynamic method for measuring end-expiratory lung volume in critically ill patients
Source: Crit Care. 2024 Apr 30;28:142. doi: 10.1186/s13054-024-04928-w (PMC11059761; doi:10.1186/s13054-024-04928-w)
Supplement: Supplementary file 1 — Additional file 1. Supplementary materials and figures. [file 13054_2024_4928_MOESM1_ESM.docx]

**Supplemental material for:**

**Clinical validation of a capnodynamic method for measuring EELV in critically ill patients.**

Sanchez Giralt JA^1^, Tusman G^2^, Wallin M^3^, Hallback M^4^, Perez Lucendo A^1^; Sanchez Galindo M^1^, Abad B, Paz Calzada E, Garcia GarciaP, Rodriguez-Huertas D, Canabal Berlanga A^1^, Suarez-Sipmann F^1,5,6^

**Rationale for the correction of the Capnodynamic method**

The overestimation of lung volume in which any CO_2_ based method systematically incurs has been well described in the past (1–3). The rationale explaining potential causes of this overestimation can be found in previous physiological studies analyzing alveolar and lung tissue CO_2_ dynamics that can be summarized as follows:

1. Dubois and Fenn studied alveolar CO_2_ dynamics during alveolar ventilation and its response to inspiratory and expiratory holds (4–7). They introduced the concept of “Equivalent lung volume” (EVL) to describe the global lung volume that contains CO_2_ and through which it diffuses and equilibrates. It includes not only the end-expiratory lung volume (i.e FRC) but also an additional volume corresponding to lung tissue and its blood content. This was inferred by the observation that, as opposed to the linear and constant decline of the alveolar partial pressure of oxygen during inspiratory/expiratory pauses, the alveolar partial pressure of CO_2_ (PACO_2_), tends to level-off as a result of the gradual equilibration between alveolar and tissue CO_2_. In addition, during an inspiratory hold, CO_2_ declines but to a level always higher than predicted due to the continuous transfer of tissue CO_2_ to the alveolar compartment. This results in a damping effect on alveolar CO_2_ fluctuations and thus on PACO_2_ levels. In other words, to maintain PACO_2_ in a stable range, a certain amount of CO_2_ constantly exits and enters the pulmonary alveolar and tissue compartments.

The measurements performed in these studies established an average relationship between the alveolar volume (i.e FRC) and ELV of 0.83 (ranging between 0.51 and 1.39) in young healthy, spontaneously breathing subjects (5).

We found a EELVCO_2_/EELVCT relationship of 0.8 ± 0.2 (Figure S1) in the current studied patients, similar to the one described for the FRC/ELV relationship, so that EELVCT was found to be on average an 80% of the EELVCO_2_ obtained value.

1. To better understand the contribution of CO_2_ tissue diffusion to the alveolar space a number of studies have attempted to quantify the amount of CO_2_ stored in lung tissue at a given moment. Obtained experimental values have varied between 0.23 and 0.38 ml of CO_2_ per 100 gr of lung tissue for each mmHg increase in PACO_2_ (6, 8–10). Although these studies clearly support the role of lung tissue CO_2_ stores it is difficult to use their estimations to approximate the true values in ventilated patients. First, data were obtained from healthy spontaneously-breathing subjects in experimental conditions. Second, it is unknown which and to what extent the whole lung parenchyma or only normally ventilated and perfused regions act as CO_2_ exchanging compartments. The difference can be significant due to the frequent heterogeneous distribution of ventilation and perfusion in mechanically ventilated lungs especially in pathological conditions. In this respect we found that the smaller the lung volume, corresponding to lungs with a larger proportion of poorly and non-aerated lung compartments, the larger the overestimation of EELVCT by EELVCO_2._ (Figures S1 and S2). We also observed that the increase in the lung gas-tissue ratio was related to a lower overestimation reinforcing the potential role of CO_2_ tissue stores in the performance of the method.

1. The capnodynamic method assumes a mass balance in which the amount of CO_2_ transferred from the blood to the alveolar compartment equals the amount transferred from the alveolar compartment to the atmosphere. However, since an equilibration between the tissue stores and alveolar CO_2_ occurs, the CO_2_ elimination (VCO_2_) measured at the airway opening may not exactly reflect the “true” VCO_2_ obtained from blood-gas diffusion through the alveolo-capillary membrane. This has led several authors to propose a correction factor to estimate alveolar VCO_2_ when CO_2_ kinetics-based methods are used to calculate lung volume or cardiac output (11–14).

Taking into account the above explanations it seems justified to apply a 20% correction to the EELVCO_2_ values in order to estimate EELV in mechanically ventilated patients. We found that such a correction significantly improved the accuracy of the method in the studied population. Such a correction however, being an estimated average, will not satisfy all possible conditions due to the inter and intraindividual expected variability of the exposed factors, leading to potential over- or under-corrections in certain extreme circumstances.

Uncorrected EELVCO_2_ values may however have an interest in itself as a monitoring parameter reflecting and including this variability in lung CO_2_ stores and dynamics that can be affected by the lung condition and the ventilator settings.

**Comparison of ARDS vs Non-ARDS patients**

The patient population finally included allowed us to compare the performance of the method in ARDS and non-ARDS patients. Regarding the loss of lung volume both CT and EELVCO_2_ showed a similar but important decrease in both non-ARDS and ARDS patients (Figure S4) compared with the theoretical predicted values for the supine position. Surprisingly EELV was only slightly lower in ARDS patients, maybe related to the fact that all except one had a COVID-19 related ARDS which have a relatively preserved lung volume.

A priori we expected a worse performance in ARDS patients due to the more severe impairment in gas exchange, larger shunt and dead space as the physiological data demonstrated. However, we did not find any major differences in the performance. Figures S5 to S8 illustrate the agreement between corrected and uncorrected values and the functional CT volume in non-ARDS and ARDS patients. Differences are small but illustrative of the compromises incurred when applying a fixed correction factor. This is seen in Non-ARDS patients where the applied correction factor tended to over-correct the method resulting in lover EELVCO_2_ values than EELVCT.

**Continuous EELV measurement**

Although the trending ability of the method was not the scope of this validation one of the major features of this methods is the continuous measurement of EELV as shown in Figure S9. A 20 min recording period is presented together with the continuous estimation of the error function of the measurement which is maintained at a very low level ensuring the quality of the measurement which average was then used for comparison with the CT obtained volume. Notice that despite a high-quality low-error recording there is an intrinsic variability in EELV of 200 to 400ml (Figure S9). As referred to in the discussion this may well be a true variability of this volume occurring in real life conditions that may have influenced the comparison with one single measurement of CT volume. A different situation is seen in figure S10 where a low-quality signal period is presented. These periods of high error measurement were excluded from the analysis.

**Dead-Space measurement**

In table 2 data related to Bohr and Enghoff dead space (VD) are reported.

With VD_Bohr_ we refer to the original dead-space formula proposed by Christian Bohr. In this formula the mean alveolar partial pressure of CO2 obtained from the mid-portion of phase III of the capnogram is used to compute VD:

VT

VD_Bohr_

PACO_2_ – PECO_2_

PACO_2_

=

Where PACO_2_ is the mean alveolar partial pressure of CO_2_ and PECO_2_ the mixed expired partial pressure of CO_2_

In Enghoff’s dead space (VDEng) , or gas exchange index, alveolar CO2 is substituted by the arterial partial pressure of CO_2_ (Pa CO_2_).

VT

VD_Eng_

PaCO_2_ – PECO_2_

PaCO_2_

=

This change implies that VD_Eng_ calculation also includes all gas exchanging units with a low ventilation perfusion ratio and shunt that also impair CO_2_ transfer thus overestimating pure dead space, the so called shunt contamination of dead space.

Interestingly the difference between VD_Eng_ and VD_Bohr_ can provide a rough estimate of the existing shunt which is relevant to the method at hand.

**Supplemental Figures**

**Figure S1:** Ratio between EELVCT and uncorrected EELVCO_2_. A ratio < 1 indicates an overestimation of the capnodynamic method and > an underestimation. The global mean is 0.81 confirming the tendency of the capnodynamic method to overestimate the measured volume. Notice the slope of the regression line (R^2^ = 0.16) indicating a trend for an overestimation at lower lung volumes and an underestimation at higher lung volumes.


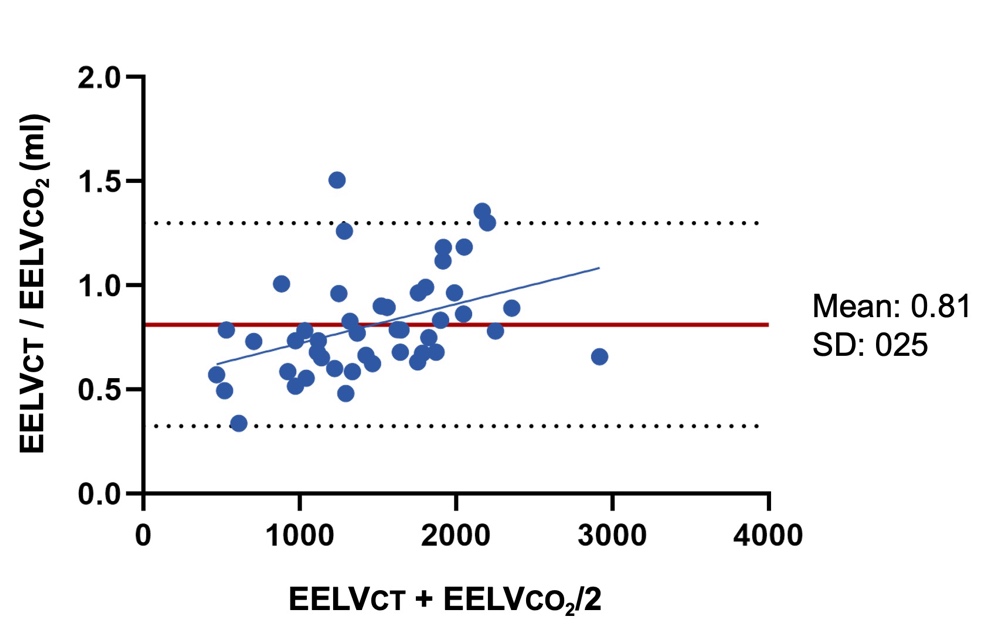


**Figure S2:** Relation between the amount of lung tissue and the percentage difference between the methods. Increasing values of gas/tissue ratio results in lower overestimations.

**
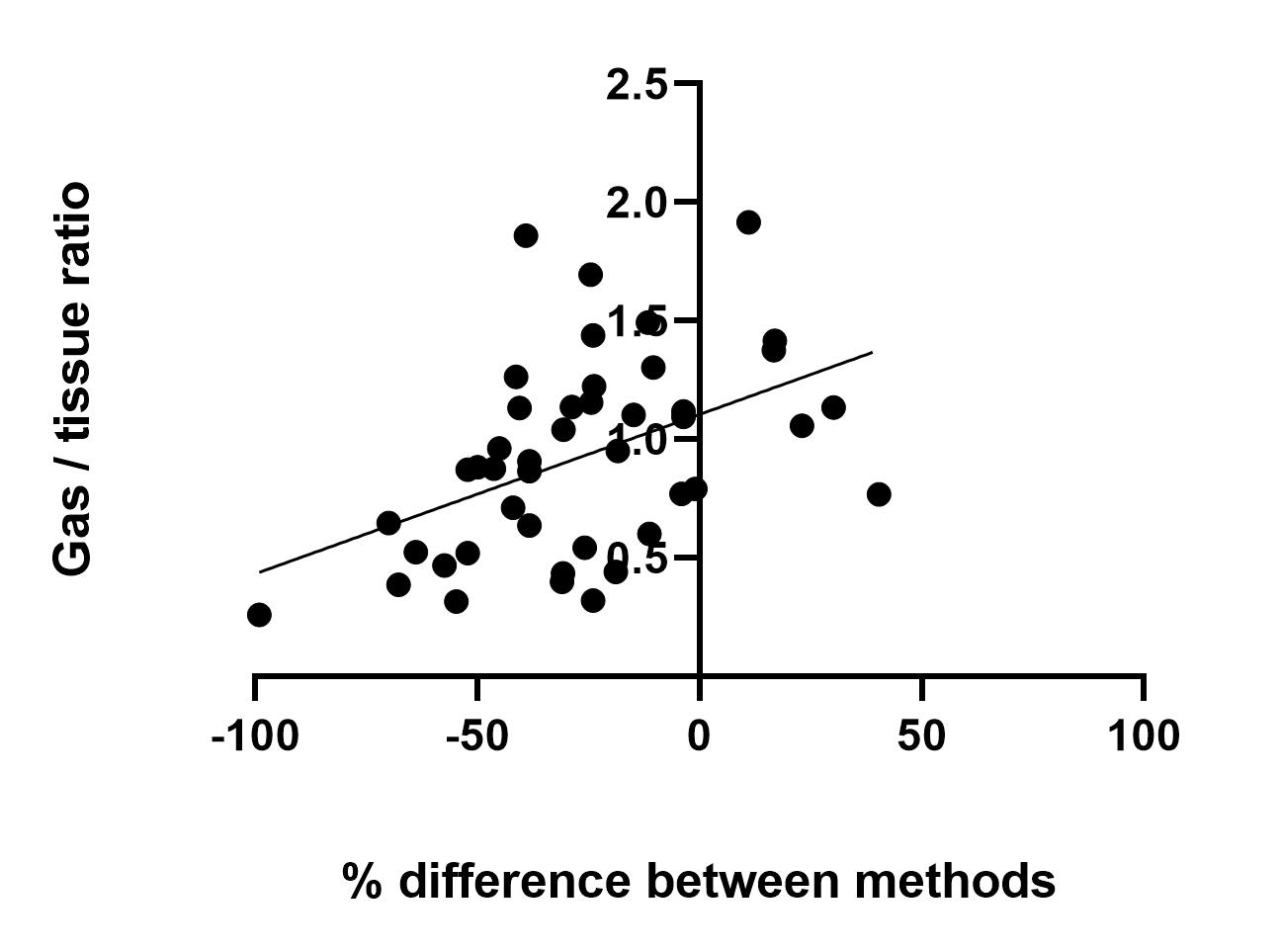
**

**Figure S3:** This overestimation was also confirmed when analyzing the residuals of the regression between EELVCO_2_ and functional EELVCT (-1000 to – 200 HU):


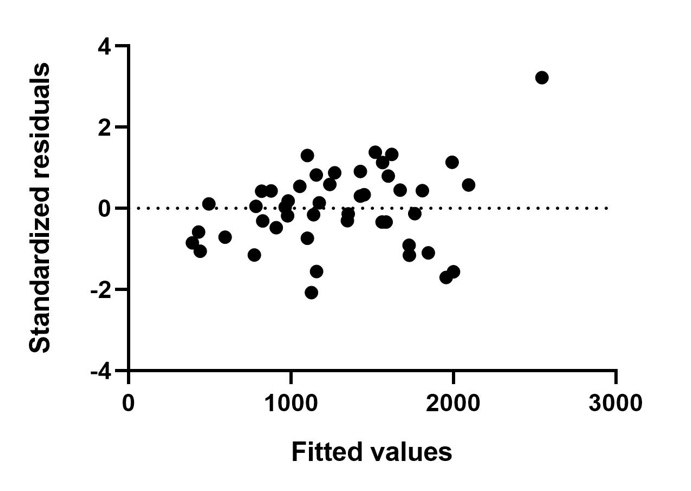


**Figure S4** presents a more detailed difference of the theoretical EELV and the obtained EELVCO_2_ in ARDS and non-ARDS patients.


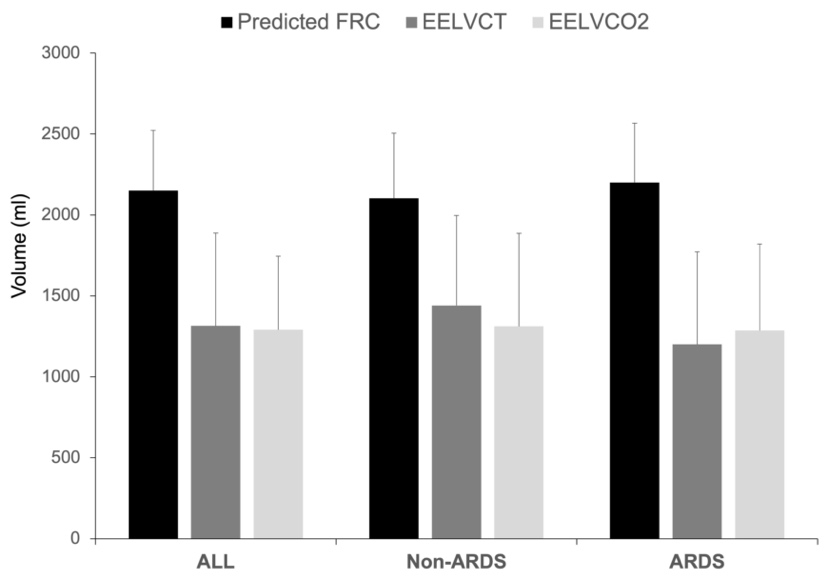


**Figure S5:** Non-ARDS patients functional EELVCT (-1000 to – 200 HU) vs uncorrected EELVCO_2_


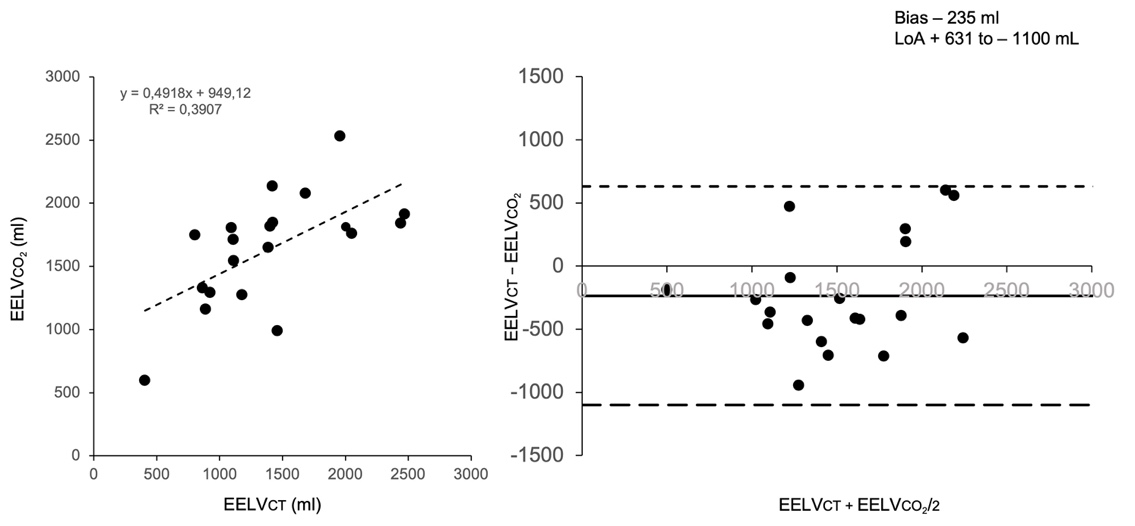


**Figure S6:** ARDS patients Functional EELVCT (-1000 to – 200 HU) vs ARDS uncorrected EELVCO_2_


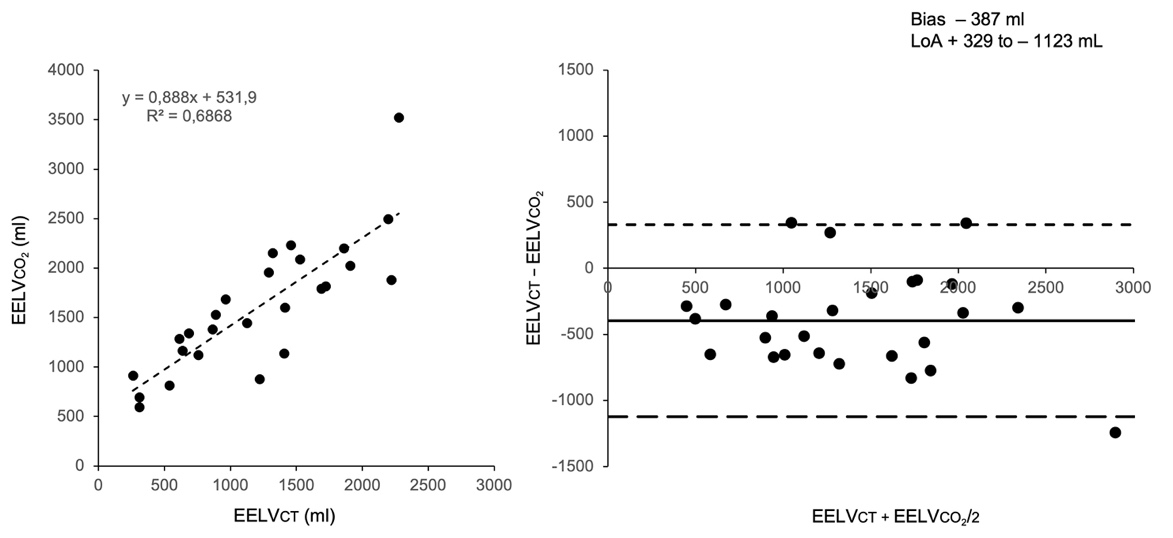


**Figure S7:** Non-ARDS patients Functional EELVCT (-1000 to – 200 HU) vs corrected EELVCO_2_


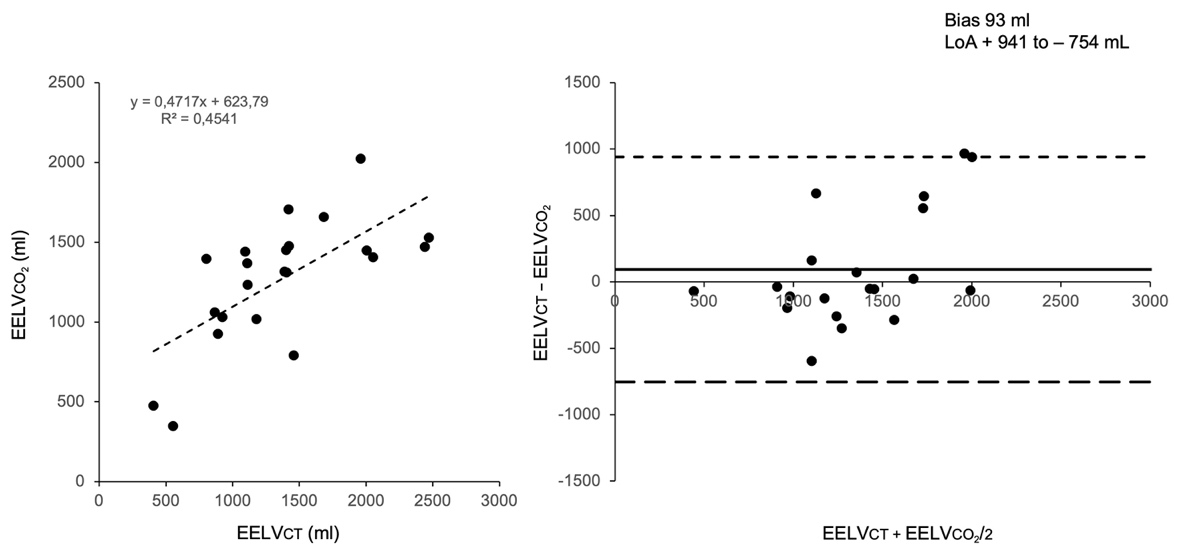


**Figure S8:** ARDS patients Functional EELVCT (-1000 to – 200 HU) vs ARDS corrected EELVCO_2_


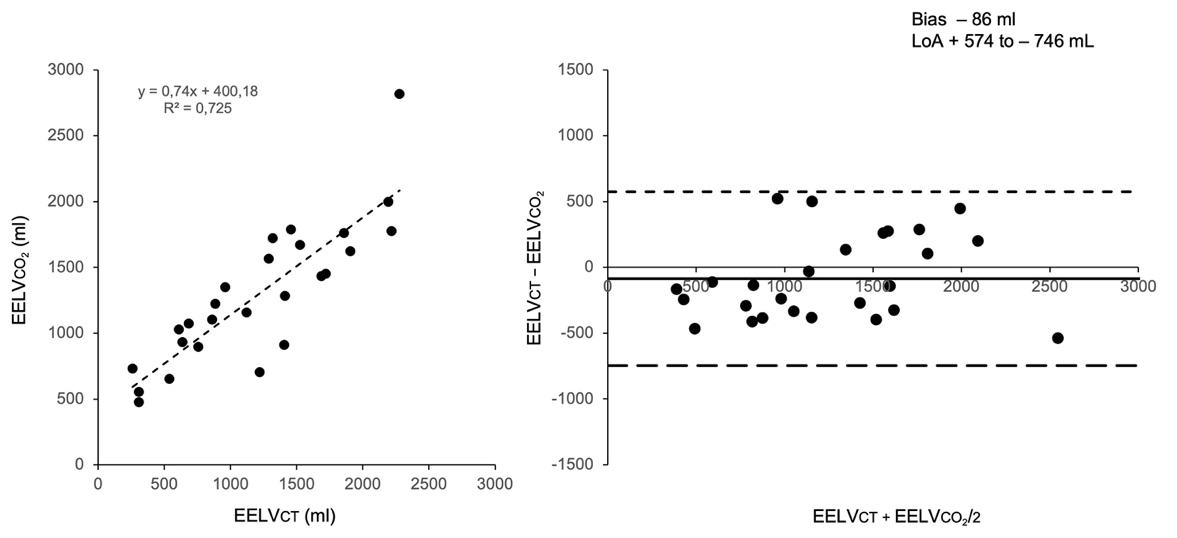


**Figure S9:** Continuous recording of EELVCO_2_ and the system error function during a 20 min period. High quality recording with a low measurement error. Notice the variability of 200 to 400 ml of EELV during the recording.


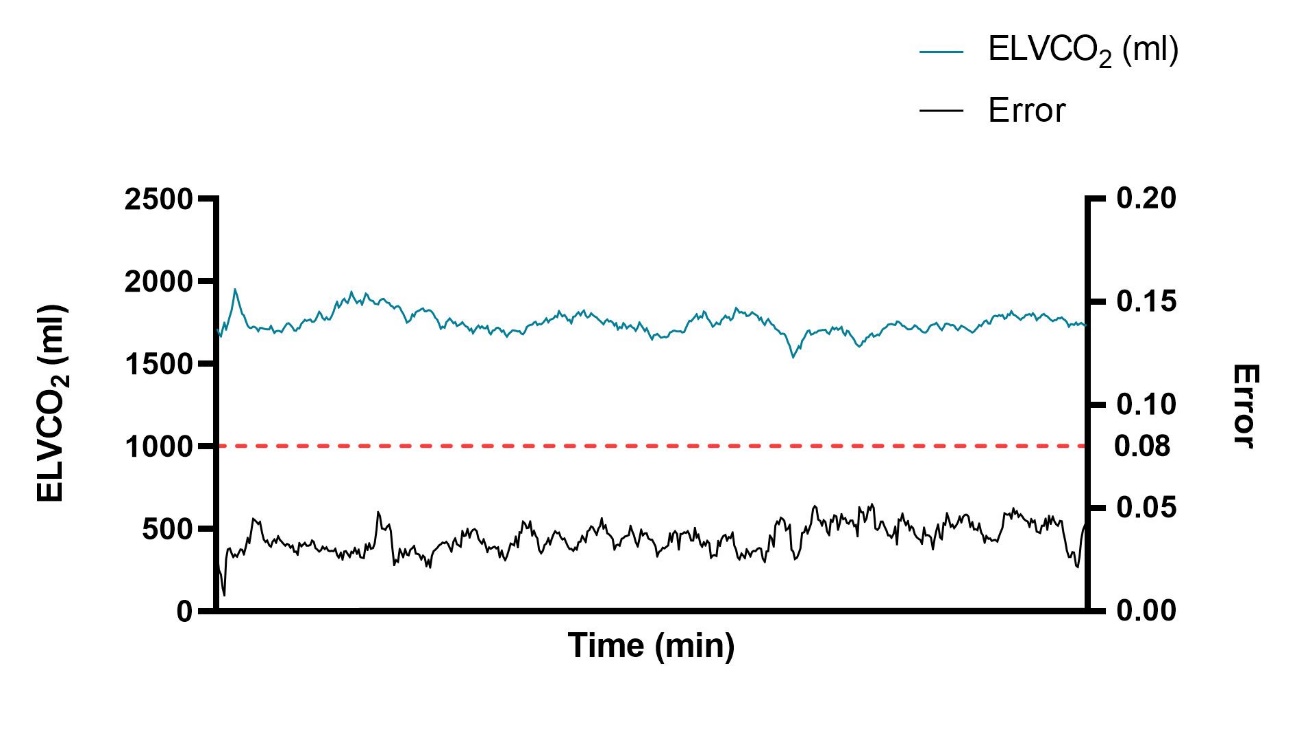


**Figure S10:** Continuous recording of EELVCO_2_ and the system error function during a 20 min period. Low quality recording with a high measurement error.


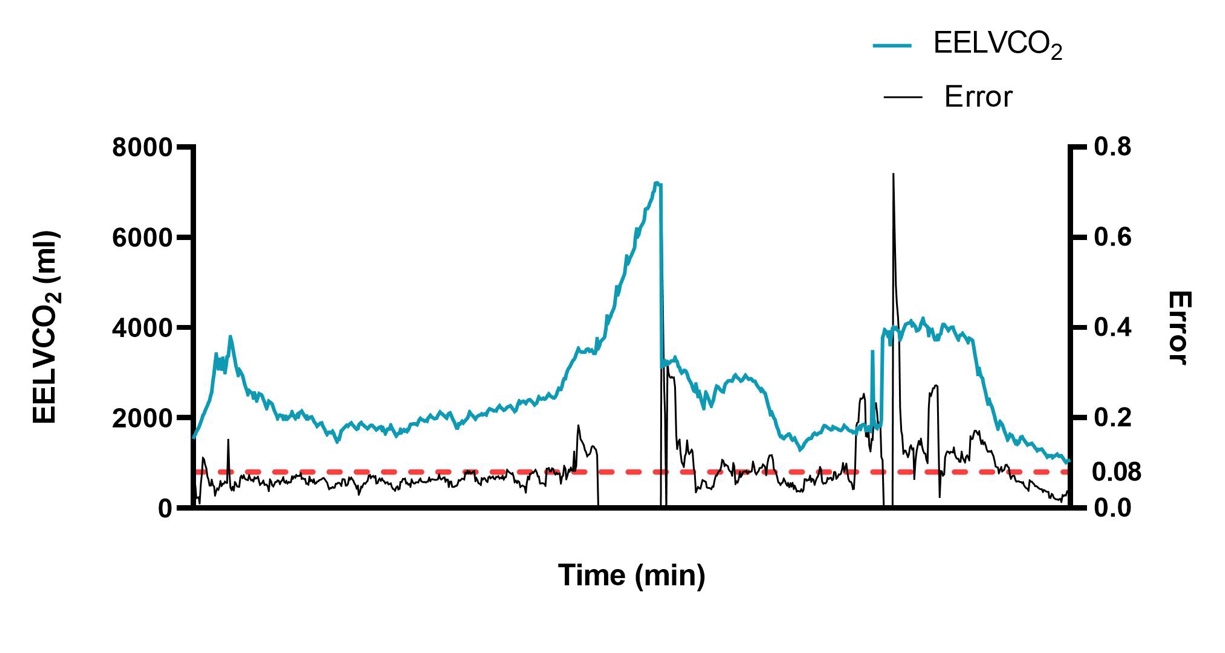


**References**

1. Brewer LM, Haryadi DG, Orr JA. Measurement of functional residual capacity of the lung by partial CO2 rebreathing method during acute lung injury in animals. *Respir care* 2007;52:1480–1489.

2. Brewer L, Orr J, Fulcher E, Markewitz B. Evaluation of a CO2 partial rebreathing functional residual capacity measurement method for use during mechanical ventilation. *J Clin Monitor Comput* 2011;25:397–404.

3. Gedeon A, Krill P, Osterlund B. Pulmonary blood flow (cardiac output) and the effective lung volume determined from a short breath hold using the differential Fick method. *J Clin Monitor Comput* 2002;17:313–321.

4. AB D, Britt A, Fenn W. Alveolar CO2 during the respiratory cycle. *J Appl Physiol* 1952;4:535–548.

5. DuBois A. Alveolar CO2 and O2 during breath holding, Expiration and inspiration. *J Appl Physiol* 1952;5:1–12.

6. DUBOIS AB, FENN WO, BRITT AG. CO2 dissociation curve of lung tissue. *J Appl Physiol* 1952;5:13–16.

7. Fenn WO, Dejours P. Composition of Alveolar Air During Breath Holding With and Without Prior Inhalation of Oxygen and Carbon Dioxide. *J Appl Physiol* 1954;7:313–319.

8. Sackner MA, Feisal KA, DuBois AB. Determination of tissue volume and carbon dioxide dissociation slope of the lungs in man. *J Appl Physiol* 1964;19:374–380.

9. Plewes JL, Olszowka AJ, Farhi LE. Amount and rates of CO2 storage in lung tissue. *Respir Physiol* 1976;28:359–369.

10. Hyde RW, Puy RJM, Raub WF, Forster RE. Rate of disappearance of labeled carbon dioxide from the lungs of humans during breath holding: a method for studying the dynamics of pulmonary CO2 exchange. *J Clin Invest* 1968;47:1535–1552.

11. Sherrill DL, Dietrich BH, Swanson GD. On the estimation of pulmonary blood flow from CO2 production time series. *Comput Biomed Res* 1988;21:503–511.

12. Preiss DA, Azami T, Urman RD. Variations in Respiratory Excretion of Carbon Dioxide Can Be Used to Calculate Pulmonary Blood Flow. *J Clin Med Res* 2015;7:83–90.

13. Beaver WL, Lamarra N, Wasserman K. Breath-by-breath measurement of true alveolar gas exchange. *J Appl Physiol* 1981;51:1662–1675.

14. Wessel HU, Stout RL, Bastanier CK, Paul MH. Breath-by-breath variation of FRC: effect on VO2 and VCO2 measured at the mouth. *J Appl Physiol* 1979;46:1122–1126.
